# Cas9-mediated excision of *Nematostella brachyury* disrupts endoderm development, pharynx formation and oral-aboral patterning

**DOI:** 10.1242/dev.145839

**Published:** 2017-08-15

**Authors:** Marc D. Servetnick, Bailey Steinworth, Leslie S. Babonis, David Simmons, Miguel Salinas-Saavedra, Mark Q. Martindale

**Affiliations:** 1 Division of Biological Sciences, University of Washington Bothell, Bothell, WA 98011, USA; 2 Whitney Laboratory for Marine Bioscience, University of Florida, St Augustine, FL 32080, USA

**Keywords:** *Nematostella*, Cnidarian, *Brachyury*, Mesoderm, Endoderm, Pharynx

## Abstract

The mesoderm is a key novelty in animal evolution, although we understand little of how the mesoderm arose. *brachyury*, the founding member of the T-box gene family, is a key gene in chordate mesoderm development. However, the *brachyury* gene was present in the common ancestor of fungi and animals long before mesoderm appeared. To explore ancestral roles of *brachyury* prior to the evolution of definitive mesoderm, we excised the gene using CRISPR/Cas9 in the diploblastic cnidarian *Nematostella vectensis*. *Nvbrachyury* is normally expressed in precursors of the pharynx, which separates endoderm from ectoderm. In knockout embryos, the pharynx does not form, embryos fail to elongate, and endoderm organization, ectodermal cell polarity and patterning along the oral-aboral axis are disrupted. Expression of many genes both inside and outside the *Nvbrachyury* expression domain is affected, including downregulation of Wnt genes at the oral pole. Our results point to an ancient role for *brachyury* in morphogenesis, cell polarity and the patterning of both ectodermal and endodermal derivatives along the primary body axis.

## Abstract (precis)

**Summary:** Brachyury is a key regulator of mesoderm, but is also found in diploblasts, which lack this tissue layer. Knockout experiments reveal its widespread effects on the endomesodermal gene regulatory network.

## INTRODUCTION

Bilaterian embryos consist of three germ layers (ectoderm, mesoderm and endoderm) and have both anterior-posterior and dorsal-ventral axes; bilaterians are thought to have evolved from a diploblastic ancestor that lacked mesoderm. Thus, the origin of mesoderm may have facilitated the evolution of more complex body plans (see Martindale and Hejnol, 2009). Despite the importance of these events in metazoan evolution, the origins of mesoderm remain obscure. Here, we approach this issue by examining the role of a gene central to chordate mesoderm development, *brachyury*, in the diploblastic cnidarian *Nematostella vectensis*.

**Fig. 1.**
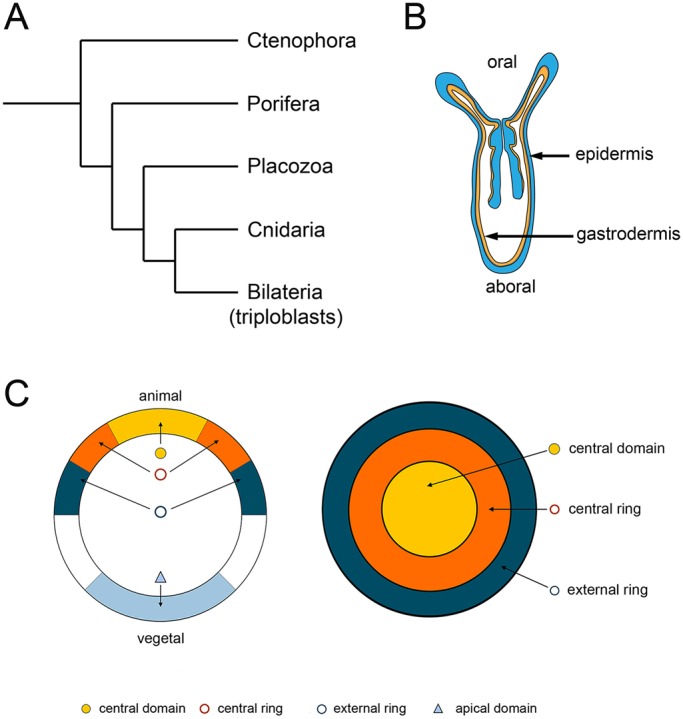
***Nematostella* oral-aboral axis and gene expression domains.** (A) Evolutionary relationships among metazoa (Dunn et al., 2014). (B) A *Nematostella* juvenile, showing the oral-aboral axis and tissue layers. (C) Gene expression domains as defined by Röttinger et al. (2012): lateral view on the left; oral view on the right.

Cnidarians are the sister group to the bilateria (Fig. 1A); the two groups diverged at least 580 million years ago (Chen et al., 2002). Cnidarians have an oral-aboral axis (Fig. 1B), and although they have historically been considered to be radially symmetric, they show unambiguous signs of bilaterality (reviewed by Rentzsch and Technau, 2016). Cnidarians have only two clearly defined germ layers, an outer ectoderm and the inner endoderm or gastrodermis that lines the gut cavity (Fig. 1B), so they are especially well suited to studies on the evolutionary antecedents of mesoderm (Martindale et al., 2004; Technau, 2001; Technau and Steele, 2011). The cnidarian gastrodermis is sometimes referred to as a bifunctional endomesodermal tissue layer because it expresses not only genes associated with endoderm development, but also several genes associated with bilaterian mesoderm development (see Technau and Steele, 2011).

Among the genes with important roles in chordate mesoderm development are the *T-box* (Tbx) genes (reviewed by Naiche et al., 2005; Papaioannou, 2014). The founding member of the Tbx gene family, *brachyury* (*bra* or *T*), is essential for proper development of mesoderm. In mice, homozygous *T* mutants lack a notochord and posterior somites (Chesley, 1935). Knockdown of *brachyury* function leads to similar loss of posterior mesoderm in *Xenopus* (Gentsch et al., 2013) and zebrafish (Martin and Kimelman, 2008). In both *Xenopus* and urochordates, ectopic expression of *brachyury* redirects ectodermal or endodermal cells, respectively, to form mesoderm (Cunliffe and Smith, 1992; see Satoh et al., 2012). Cnidarians have clear *brachyury* orthologs (Bielen et al., 2007; Scholz and Technau, 2003; Hayward et al., 2015; Yasuoka et al., 2016). In *Nematostella vectensis* embryos, *Nvbrachyury* (*Nvbra*) is expressed in the ‘central ring’ prior to gastrulation (Fig. 1C), a region that will give rise to the pharynx (Röttinger et al., 2012). The *Nvbra* expression pattern is reminiscent of its expression around the blastopore in other animals (Technau, 2001), but, given the absence of definitive mesoderm in cnidarians, its role in diploblasts remains unclear.

To gain insight into the role of *brachyury* during embryonic development in a diploblast, we used CRISPR/Cas9 (Jinek et al., 2012) to excise the *Nvbra* gene from early *Nematostella* embryos. *Nvbra* RNA expression is effectively eliminated in most *F*_0_ embryos, as demonstrated by both qPCR and *in situ* hybridization. In the absence of *Nvbra*, embryos initiate gastrulation normally, but the pharynx fails to form, embryos do not elongate and, although endoderm is specified, its organization is badly disrupted. Furthermore, deletion of *Nvbra* has widespread effects on components of the gene regulatory network that are active at the oral pole of the embryo [termed the endomesodermal GRN (Röttinger et al., 2012)] and patterning along the oral-aboral axis.

## RESULTS

### Cas9 excises *Nvbra* in early embryos

Gene models and ESTs show that the *Nematostella vectensis brachyury* (*Nvbra*) gene consists of 7 exons, spanning about 5 kb of genomic sequence; the T-box is encoded in exons 1-4 (Fig. S1A). To disrupt the *Nvbra* gene using Cas9, we generated five guide RNAs (gRNAs) to blanket the gene (Table S1). The target sites range from 50 bp upstream of the transcription initiation site to a site within exon 6 (Fig. S1A, triangles).

Excision of *Nvbra* was most effective when all five gRNAs were injected with Cas9. We injected embryos with gRNAs 1 and 2; with gRNAs 3, 4 and 5; or with all five gRNAs, and assayed the embryos using *in situ* hybridization for *Nvbra* expression. Normal *Nvbra* expression has been described previously (Scholz and Technau, 2003; Fritzenwanker et al., 2004; Röttinger et al., 2012) and is shown in Fig. S2. Expression is undetectable during cleavage stages. By the blastula stage, *Nvbra* is expressed in scattered patches of cells; soon thereafter, expression becomes localized to a ring at the oral pole that later gives rise to the pharynx (Magie et al., 2007; Röttinger et al., 2012). As shown in Fig. S3A, 74% of embryos injected with all five gRNAs lacked detectable *Nvbra* expression by *in situ* hybridization. When only gRNAs 1 and 2 were injected, this was reduced to 66%, and, when gRNAs 3, 4 and 5 were injected, it was reduced to only 33%. Examples of mosaic embryos, presumably resulting from disruption of *Nvbra* in some, but not all, blastomeres, are shown in Fig. S3D,E. The experiments described below were conducted using all five gRNAs to disrupt the *Nvbra* gene, both to ensure a high rate of excision and, because *Nvbra* is transcribed at a high level early in development (Helm et al., 2013; Tulin et al., 2013), to excise the gene early, before any *Nvbra* transcripts are generated.

Analysis of 10 uninjected control embryos showed the expected 2.3 kb genomic band in all embryos (Fig. S1B). In contrast, none of 20 embryos injected with gRNAs 1-5 and Cas9 (which we call *Nvbra*/Cas9 embryos) showed a robust 2.3 kb band; four showed weak bands at 2.3 kb (Fig. S1C). This incomplete excision of the *Nvbra* gene in some embryos may be due to disruption of only one of the two alleles or, more likely, to mosaic excision, with the gene removed in some, but not all, blastomeres. Even in cases in which a 2.3 kb band was detected, it is possible that small deletions occurred that are not detected by PCR.

To determine whether the amplified bands in the *Nvbra*/Cas9 embryos correspond to disrupted *Nvbra* genes, we cloned three gel-purified bands (Fig. S1C-E). Sequencing showed that the DNA had been cut near the target sites for gRNAs 1 and 5. The DNA cleavage sites did not match precisely the predicted cut sites, but had short insertions or deletions at the cleavage site junctions, as observed by others (see Varshney et al., 2015).

**Fig. 2.**
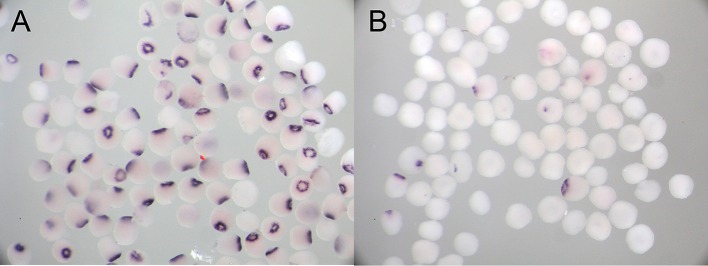
***Nvbra* expression in uninjected control and *Nvbra*/Cas9 embryos.** *In situ* hybridization showing *Nvbra* expression in (A) uninjected embryos and (B) sibling embryos injected with *Nvbra* gRNAs and Cas9 (*Nvbra*/Cas9 embryos). Most control embryos show characteristic staining around the oral pole. Most *Nvbra*/Cas9 embryos show no staining; some show a smaller region of staining, whereas only a few show the normal staining pattern.

To validate the results of genomic DNA analysis, we examined injected embryos and sibling controls at 48 h post-fertilization (hpf) by *in situ* hybridization. At 48 hpf, control embryos showed strong *Nvbra* staining, localized to the region surrounding the blastopore (Fig. 2A): 89.7% of embryos showed this pattern; 5.2% showed weaker, but still detectable, polarized staining; and 5.2% showed no detectable staining (*n*=58). In contrast, *Nvbra*/Cas9 embryos showed very little staining: 83.6% showed no detectable signal; 11.5% showed a reduced signal; and only 4.9% showed the wild-type pattern (*n*=61, Fig. 2B). The weakly staining group may include embryos in which the *Nvbra* gene was incompletely excised (i.e. part of the gene might remain), or in which the gene was excised in some, but not all, cells, generating mosaic embryos. The strong reduction in *Nvbra* gene expression relative to controls is congruent with the DNA analyses; together, these lines of evidence suggest that the gene has been disrupted in the vast majority of embryos.

### Excision of *Nvbra* blocks pharyngeal development

*Nvbra*/Cas9 embryos showed normal cleavage, and early gastrulation movements appeared to be unaffected. However, in surviving *Nvbra*/Cas9 embryos, it was difficult to distinguish the oral from the aboral end, and there was no apparent axial elongation. To characterize these effects in more detail, we examined embryos using immunohistochemistry and confocal microscopy.

**Fig. 3.**
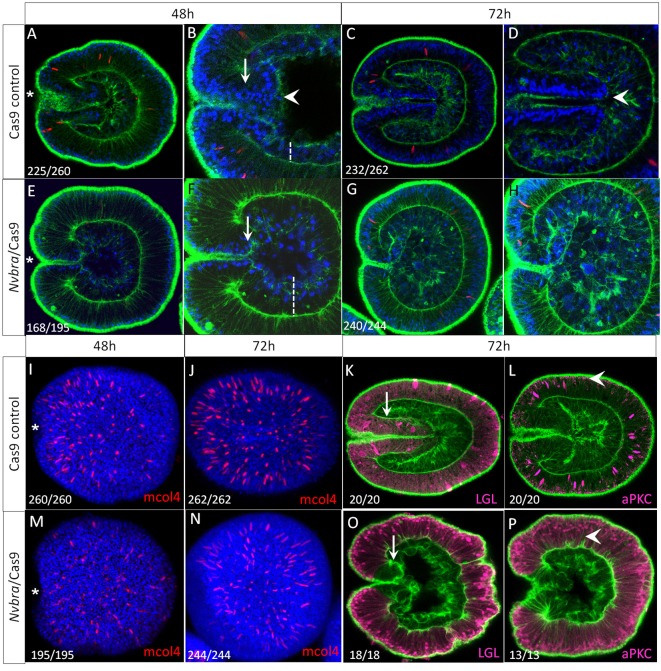
**Pharyngeal development and ectodermal cell polarity, but not cnidocyte differentiation of surface ectodermal cells, are disrupted after *Nvbra* excision.** (A-D) Control embryos were injected with Cas9 only. (A,B) At 48 h post-fertilization (hpf) the blastopore is visible (arrow) and a well-defined epithelial endoderm has formed (dotted line). Ectodermal cells protrude into the archenteron to form the pharynx rudiment (arrowhead). (C,D) By 72 hpf the ectoderm has extended well into the blastocoel (arrowhead). (E-H) In *Nvbra*/Cas9 embryos, the pharynx fails to form. (E,F) At 48 hpf, the blastopore is visible (arrow) but the endoderm is thicker than in controls (compare dotted lines in B and F) and appears disorganized. No pharyngeal ectoderm extends into the archenteron. (G,H) By 72 hpf, the blastopore is still evident but no pharynx has formed. The endoderm is highly disorganized with cells filling the blastocoel, and embryos appear rounded when compared with the elongated phenotype of control embryos. (I-L) Cas9 control embryos develop cnidocytes normally. At 48 hpf (I) and 72 hpf (J), cnidocytes (red, anti-mcol4) are abundant throughout the ectoderm. Control embryos (72 hpf) also exhibit ectodermal expression of (K) Lgl and highly polarized expression of (L) aPKC (arrowhead) in the apical cortex of ectodermal cells. (M-P) Cnidocytes are present in the ectoderm of *Nvbra*/Cas9 embryos at both (M) 48 hpf and (N) 72 hpf. (O) Lgl is still basolateral and restricted to the ectodermal cells; there is abrupt cessation of Lgl staining at the blastopore (arrow, compare with K). Expression of aPKC (P) is no longer apically restricted, spreading into the basal regions of ectodermal cells (arrowhead). Ratios in the bottom left corner indicate the number of embryos showing the indicated phenotype (wild type or abnormal)/total number of embryos counted; only embryos with proper ectoderm formation were counted in order to exclude dead or clearly abnormal embryos. Phenotypes of 72 hpf *Nvbra*/Cas9 embryos are quantified in more detail in Fig. S5. The blastopore is on the left of each image, indicated by an asterisk in the first column. Images A-H and K,L,O,P are single optical sections; images I,J,M,N are 3D rendered from *z*-stacks.

*Nvbra*/Cas9 embryos were collected at 48, 72 and 96 hpf, and processed for immunohistochemistry. We injected separate samples with Cas9 protein alone (Cas9-only) or *Nvbra* gRNAs alone (gRNA-only). Both controls were indistinguishable from uninjected embryos; Fig. 3 shows Cas9-only controls with *Nvbra*/Cas9 embryos. (Uninjected embryos and gRNA-only controls are shown in Fig. S4.)

The onset of gastrulation occurs well before 48 hpf in both control and *Nvbra*/Cas9 embryos, and invagination results in the formation of an epithelial endoderm in both sets of embryos (Fig. 3A,B,E,F). In control embryos, endoderm formation is followed by the onset of pharyngeal development; by 72 hpf, control embryos have a rudimentary pharynx extending into the archenteron and embryos are elongated along the oral-aboral axis (Fig. 3A-D). In contrast, *Nvbra*/Cas9 embryos undergo gastrulation and initial endoderm development (Fig. 3E,F) but pharyngeal ectoderm does not form and the endodermal layer is thicker and appears less well-organized compared with controls. By 72 hpf four phenotypes associated with *Nvbra* excision were observed. A small number of embryos appeared to be unaffected; all remaining embryos completely lack pharyngeal development (Fig. S5). Most *Nvbra*/Cas9 embryos (82%) have highly disorganized non-epithelial endoderm that fills the blastocoel (Fig. 3G,H). Although the blastopore is visible, no pharynx can be observed. By 96 hpf, few embryos were still alive, and those that were appeared to be undergoing epithelial degeneration in both germ layers (Fig. S6).

### Excision of *Nvbra* disrupts ectodermal polarity but not cell specification

To determine whether *Nvbra* excision affects other cell types, we examined two ectodermal cell markers. First, we assayed for the presence of developing cnidocytes (cnidarian-specific stinging cells). Cnidocytes develop early (48 hpf) in ectoderm and can be detected using an antibody against the cnidocyte-specific protein minicollagen 4 (Zenkert et al., 2011; Babonis et al., 2016). Both control and *Nvbra*/Cas9 embryos had abundant cnidocytes throughout the ectoderm at 48 and 72 hpf (Fig. 3I,J,M,N), demonstrating that development of this cell type is unaffected by the absence of *Nvbra*.

We also examined the expression of two proteins associated with epithelial cell polarity: lethal giant larvae (Lgl) and atypical protein kinase C (aPKC) (Salinas-Saavedra et al., 2015). In control embryos, Lgl is restricted to the ectoderm (including the developing pharyngeal ectoderm) and is expressed from the basal to the apical membrane of ectodermal cells whereas aPKC is restricted to only the apical region of surface ectodermal cells (Fig. 3K,L). In *Nvbra*/Cas9 embryos, the absence of pharyngeal development is accompanied by an abrupt cessation of Lgl expression at the blastopore; expression does not extend into cells of the archenteron (Fig. 3O). aPKC expression expands into the basal regions of the cells (Fig. 3P), indicating that ectodermal cell polarity is affected by *Nvbra* excision.

### *Nvbra* affects gene expression both within and outside its expression domain

**Fig. 4.**
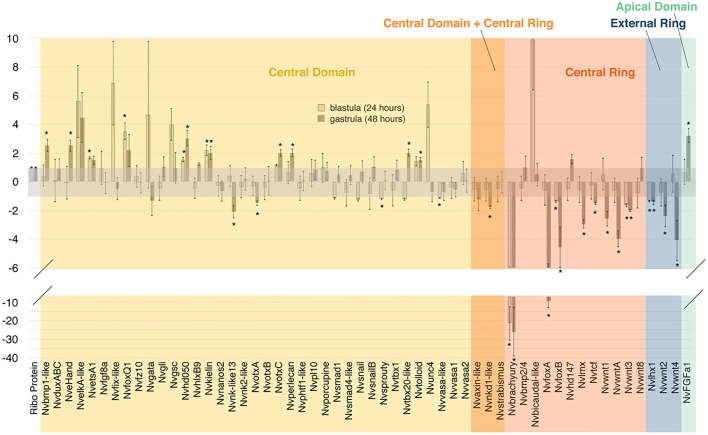
**qPCR of genes of the blastoporal gene regulatory network.** Bars indicate relative levels of expression of genes at 24 and 48 hpf. Samples were normalized to ribosomal protein P0. Reductions in expression are shown as the negative reciprocal of the expression level, in order to facilitate visualization. Asterisks indicate significant differences (*P<0.05).

To test the effects of *Nvbra* excision on gene expression, we isolated RNA from uninjected and *Nvbra*/Cas9 embryos at 24 hpf (blastula) and 48 hpf (gastrula), and analyzed it by qPCR. We quantified relative levels of expression of a panel of 60 target genes (Fig. 4); most of these were identified as potential components of the ‘endomesodermal’ gene regulatory network (Röttinger et al., 2012) in *Nematostella*. The genes showing the strongest reduction in expression are *Nvbra* itself (confirming the efficacy of the Cas9 excision) and *NvfoxA.* We estimated by DNA analysis that >80% of embryos showed excision of the *Nvbra* gene, whereas qPCR shows a reduction of more than 95% at the blastula stage. The most likely explanation for this apparent discrepancy is that even those embryos that retain a copy of the gene may have only a single copy per cell, or they are mosaic embryos (retaining a copy in only a few cells). So, even in embryos that retain a detectable copy of the gene, expression is likely to be significantly reduced.

Röttinger et al. (2012) described several regions of gene synexpression in 24 hpf embryos: the central domain includes the animal pole; the central ring (where *Nvbra* is expressed) surrounds the central domain; the external ring surrounds the central ring (Fig. 1C); and the apical domain is at the aboral pole. After *Nvbra* excision, most central ring genes we examined show strongly reduced expression (see Fig. 4). Because they are expressed in the same region as *Nvbra*, this suggests that at least some of these genes may be direct *Nvbra* transcriptional targets. In contrast, expression of many genes in the central domain (which gives rise to the endoderm) is increased; only a few are reduced, and many show little or no significant difference in expression (Fig. 4). These results show that *Nvbra* normally affects gene expression in cells several cell diameters from the central ring. As Nvbra is a transcription factor and acts cell-autonomously, the effects on cells in other domains are presumably mediated by signals, probably members of the Wnt family, secreted from central ring cells.

Expression of several Wnt gene family members is strongly reduced by *Nvbra* excision. *WntA*, *Wnt1*, *Wnt3* and *Wnt8* are expressed in the central ring, whereas *Wnt2* and *Wnt4* are expressed in the external ring at the blastula stage. In *Nvbra*/Cas9 embryos, expression of all Wnts except *Wnt8* is strongly reduced by the gastrula stage. *Nvtcf*, an effector of the canonical Wnt pathway, is also reduced in *Nvbra*/Cas9 embryos.

### *Nvbra* affects spatial gene expression along the oral-aboral and directive axes

**Fig. 5.**
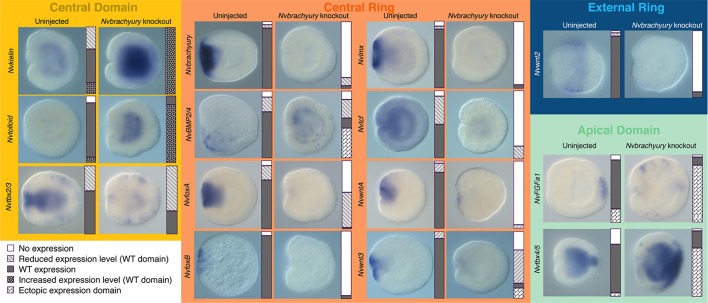
**Gene expression in control and *Nvbra*/Cas9 embryos.** *In situ* hybridization of 48 hpf control (left columns) and *Nvbra*/Cas9 embryos (right columns). In all images, oral is towards the left. Genes are organized according to their normal expression domain at 24 hpf (Fig. 1C). Bars to the right of each figure indicate the proportion of each phenotype observed (see key). Numbers of embryos scored for each panel are in Table S2.

To learn more about the spatial expression patterns of genes affected by *Nvbra* excision, and to validate the results of qPCR analysis, we assayed the expression of 14 genes by *in situ* hybridization in 48 hpf *Nvbra*/Cas9 embryos and uninjected controls (Fig. 5). Data are arranged according to the domain of expression of each gene, when known (Röttinger et al., 2012).

Two genes expressed in the central domain at 24 hpf, *NvKielin* and *NvTolloid*, are upregulated in *Nvbra*/Cas9 embryos, consistent with our qPCR results; both normally modulate BMP signaling. *Nvtbx2/3* is expressed both at the oral pole and in invaginating endoderm, and in scattered ectodermal cells (M.D.S. and M.Q.M., unpublished). Interestingly, in *Nvbra*/Cas9 embryos, oral and endodermal *Nvtbx2/3* expression are strongly reduced, whereas ectodermal expression appears to be unaffected.

Within the central ring, where *Nvbra* is expressed, most genes we examined were downregulated in *Nvbra*/Cas9 embryos. *NvfoxA* is normally expressed strongly in the central ring (Martindale et al., 2004; Fritzenwanker et al., 2004; Magie et al., 2007; Röttinger et al., 2012). Most control embryos (75/105) showed strong *NvfoxA* expression, but very few *Nvbra*/Cas9 embryos (4/195) showed normal expression (100/195 reduced expression, 91/195 no expression) (Fig. 5). The reduction in *NvfoxA* expression is again consistent with qPCR data.

*NvBMP2/4* (*Nvdpp*) is expressed in the central ring; at 48 hpf this expression is asymmetric, localized to one side of the directive axis (Finnerty et al., 2004; Röttinger et al., 2012; Matus et al., 2006; Saina et al., 2009). Most control embryos (38/55, 69%) showed this asymmetric expression pattern. In nearly half of *Nvbra*/Cas9 embryos (28/62, 45%) the expression pattern was radialized. Finally, although *Nvwnt2* is expressed in the external ring at the blastula stage, by 48 hpf it is expressed about halfway between the two poles; its expression is sharply reduced in *Nvbra*/Cas9 embryos.

The other central ring genes we examined (Fig. 5) showed reduced expression after *Nvbra* excision. Together, the qPCR and *in situ* hybridization data indicate that excision of *Nvbra* leads to strong disruption of expression of many genes at the oral pole of the embryo. Most central ring genes that we examined showed strongly reduced expression, suggesting that *Nvbra* normally acts, directly or indirectly, to activate genes in its expression domain.

We wished to determine whether expression of genes at the opposite, aboral, pole would also be disrupted. *NvFGFa1* and *Nvtbx4/5* are normally expressed at the aboral pole, and are part of the pathway leading to development of the ciliary apical organ (Matus et al., 2007; Rentzsch et al., 2008; Sinigaglia et al., 2013, 2015). In most control embryos (77/108, 71%), *NvFGFa1* is expressed in a small patch at the aboral pole (20% show an expanded patch of expression; 8% show no expression) (Fig. 5). In *Nvbra*/Cas9 embryos, the *NvFGFa1* expression domain was expanded: 24/201 (12%) embryos showed normal expression, but 169/201 (84%) showed expanded expression, with patches of expression far from the aboral pole. Expression of *Nvtbx4/5* was similarly affected. Most control embryos (45/64, 70%) showed expression in a small spot at the aboral pole (6% show a broader distribution, 23% show no expression). In *Nvbra*/Cas9 embryos, most (35/49, 84%) show an expanded expression domain (Fig. 5).

In summary, in *Nvbra*/Cas9 embryos, expression of two aboral genes is delocalized. This suggests that *Nvbra* is part of a regulatory pathway at the oral pole that constrains expression of *NvFGFa1*, *Nvtbx4/5* and presumably other genes, to the apical domain at the aboral pole; in the absence of *Nvbra*, that constraint is lifted and aboral genes show expanded expression.

## DISCUSSION

### Cas9-mediated excision of the *Nvbra* gene

We used CRISPR/Cas9 to disrupt the *Nvbra* gene in early *Nematostella* embryos. Using a multiple gRNA approach, a high proportion of injected *F*_0_ embryos lack the normal *Nvbra* genomic DNA fragment, as assayed by PCR of individual embryos and directly confirmed by cloning the edited DNA (Fig. S1). Furthermore, most injected embryos show no detectable expression of *Nvbra* RNA by *in situ* hybridization (Fig. 2), a result confirmed by qPCR (Fig. 4). Based on our morphological observations, the proportion of embryos with disrupted *Nvbra* function is even higher, suggesting that, even when the gene appears to be present, mutations may have been introduced that disable protein function.

Our use of multiple gRNAs to target *Nvbra* is novel, and a concern raised by this approach is the possibility of Cas9-mediated cleavage of off-target sites (OTSs). Two recent studies showed that both the number and position of mismatches affect Cas9 specificity. The presence of two mismatches, especially within the PAM-proximal 12 bases, reduces Cas9 cleavage substantially, while three mismatches eliminated detectable cleavage of OTSs for most loci (Hsu et al., 2013). Similarly, Pattanayak et al. (2013) observed that cleavage of OTSs with three or more mismatches occurred at frequencies at least 100-fold lower than the target site; the sole exception was an OTS with three mismatches, only one of which was in the PAM-proximal region. Nearly all the possible OTSs in this study (see Materials and Methods) contained four mismatches; only two OTSs had three mismatches, two of which were in the PAM-proximal 12 bases. Although we cannot eliminate the possibility of off-target effects, our analysis argues that OTSs are likely to be cleaved only rarely, so we are confident that the effects we observe are attributable to excision of the *Nvbra* locus.

Even with multiple gRNAs, we see residual *Nvbra* transcription in a few cells in some embryos (Fig. 2B), suggesting that the gene has not been excised from all cells in these embryos. Similarly, although we often see excision of a large DNA fragment, there is variation in the cleavage sites (Fig. S1). These observations suggest that, although our strategy is effective, further optimization of the conditions for gene excision in *Nematostella* embryos may be possible. Interestingly, Kraus et al. (2016) used Cas9 and two gRNAs to edit the *Nematostella APC* gene; they reported only on mosaic embryos obtained.

The rate of excision of *Nvbra* that we observe is higher than that observed by Ikmi et al. (2014) in a previous report of Cas9-mediated gene editing in *Nematostella*. This could be due to several factors. First, the use of multiple gRNAs may increase the editing rate by introducing multiple cuts to genomic DNA. Second, the high transcription rate of the *Nvbra* gene at early stages of development (Helm et al., 2013; Tulin et al., 2013) could reflect a more open configuration of this chromosomal region during early stages; such an open configuration might make the gene more accessible to Cas9. [The site targeted by Ikmi et al. (2014) is not expressed until adult stages.] The results of Perez-Pinera et al. (2013) suggest that Cas9 can access transcriptionally inactive sites, but it is unclear whether this is true under all conditions. Finally, technical differences may contribute to the increased excision rate; these include different proportions of gRNA:Cas9, microinjection procedures, and different gRNA efficiencies (e.g. Gagnon et al., 2014; Varshney et al., 2015).

### Effects of *Nvbra* excision on pharyngeal development

*Nematostella* gastrulation occurs in two distinct waves. Initially, at ∼24-28 hpf (at 16°C), presumptive endodermal cells at the oral pole undergo apical constriction and the endodermal epithelium buckles inwards; the endoderm continues to move inwards powered in part by filopodial extensions to the basal surface of the overlying ectodermal epithelium (Magie et al., 2007; Tamulonis et al., 2011; Kraus and Technau, 2006). Magie et al. (2007) concluded that neither ingression nor any epithelial-to-mesenchymal transition (EMT) occurs during gastrulation in *Nematostella.* The second wave occurs during pharynx formation [∼36-60 hpf (Magie et al., 2007)] when cells of the central ring involute, invade the gastric cavity and form a tall columnar epithelium, maintaining their epithelial connection to both the inner gastrodermis and the overlying epidermis; however, little is known about the forces that drive this morphogenetic movement.

In *Nvbra*/Cas9 embryos, early gastrulation movements appear to be unaffected, though subsequent events – pharynx formation and endodermal patterning – are strongly disrupted. This suggests that *Nvbra* is not required for initial invagination of endodermal epithelium to form the blastopore. Recently, Yasuoka et al. (2016) reported very similar results; they too observed that gastrulation occurs normally, but pharynx formation is inhibited, after morpholino knockdown of *brachyury* in the coral *Acropora digitifera.* Similarly, in the ctenophore *Mnemiopsis leidyi*, gastrulation occurs after *brachyury* knockdown, but formation of the stomodeum and pharynx are disrupted (Yamada et al., 2010). In vertebrate embryos as well, initial gastrulation movements occur in the absence of *brachyury* function [Martin and Kimelman, 2008 (zebrafish); Gentsch et al., 2013 (*Xenopus*); Chesley, 1935 (mice)], but subsequent elongation of the mesoderm does not occur normally, leading to the characteristic absence of posterior mesoderm.

The reasons that the pharynx does not form in the absence of *Nvbra* are not clear, but several models are possible. First, the central ring cells, which normally express *Nvbra* and form pharyngeal ectoderm, may be respecified to form endoderm. This would result in an increase in endoderm (and an increase in expression of some endodermal genes) and a reduction in expression of many central ring genes, as we observe. Second, *Nvbra* knockout leads to reduced expression of Wnts by central ring cells (Figs 4 and 5), and inhibition of Wnt signaling leads to failure of the pharynx to form (Röttinger et al., 2012). Finally, the loss of normal ectodermal cell polarity, as evidenced by the changes in distribution of Lgl and aPKC (Fig. 3), may lead to changes in cell adhesion that prevent pharyngeal morphogenesis.

Numerous lines of evidence point to a role for *brachyury* in regulating cell adhesion and migration in other systems. In a ctenophore and a coral, *bra* inhibition blocks formation of the stomodeum/pharynx (Yamada et al., 2010; Yasuoka et al., 2016). *Drosophila bra* mutants have defects in Malpighian tubule elongation and midgut constriction (Singer et al., 1996). In ascidian *bra* mutants, the notochord fails to elongate (Chiba et al., 2009). *T/T* embryonic stem cells in chimeric mice are unable to leave the primitive streak; this appears to be due to an adhesion defect (Wilson et al., 1995). Finally, *T* overexpression leads to epithelial-mesenchymal transitions (EMT) in several human tumors (Du et al., 2014; Fernando et al., 2010; Shimoda et al., 2012), and is associated with tumor cell metastasis (Palena et al., 2014; Pinto et al., 2014; Roselli et al., 2012). In some cases, *bra* promotes EMT by repressing E-cadherin expression (Fernando et al., 2010; Sun et al., 2014). The loss of pharyngeal development in *Nvbra*/Cas9 embryos may be due in part to modification of cell adhesion in presumptive pharyngeal and/or endodermal cells (Fig. 3), which is normally mediated by *Nvbra* and its downstream targets.

Our results reveal intriguing parallels between the formation of the *Nematostella* pharynx and the chordate notochord. Notochord development requires both *brachyury* and *FoxA* [Shimauchi et al. (2001) (urochordate); Martin and Kimelman (2008) and Del-Pra et al. (2011) (zebrafish); O'Reilly et al. (1995) (*Xenopus*); Ang and Rossant (1994) and Weinstein et al. (1994) (mouse)]. In most (but not all) chordates, the notochord extends, contributing to elongation of the embryo, and secretes signals that organize surrounding tissues. In *Nematostella*, *Nvbra* and *NvfoxA* are co-expressed in the cells that give rise to the pharynx, which extend, contribute to embryo elongation, and appear to be involved in endoderm organization. Although the two structures are not homologous, *brachyury* and *FoxA* may together mediate some cellular processes that occur in both systems.

### Effects of *Nvbra* excision on endoderm

**Fig. 6.**
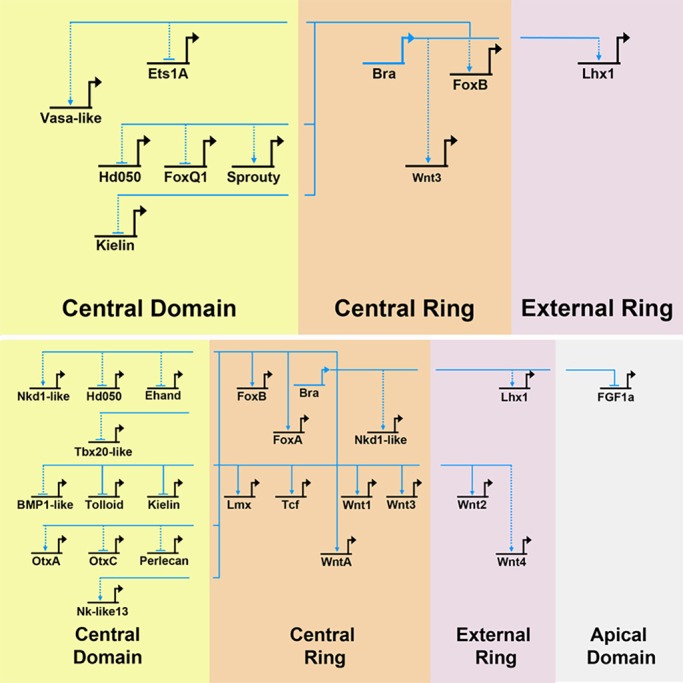
**Effects of *Nvbra* on the blastoporal gene regulatory network.** Gene regulatory relationships in the endomesodermal GRN described by Röttinger et al. (2012). Because Nvbra is a transcription factor, we assume that Nvbra can directly affect only genes in its expression domain (the central ring). Effects on domains outside the central ring are assumed to be mediated by signaling molecules; indirect effects are indicated by the broken lines between regions of the embryo. Dotted arrows indicate interactions inferred from qPCR. Solid arrows indicate interactions inferred from both qPCR and *in situ* hybridization. Genes analyzed by *in situ* hybridization alone are not included. Upper panel: 24 h. Lower panel: 48 h. Genes are arrayed according to their normal expression domain at 24 h. Genes closer to the top of the diagram are transcribed earlier in development; those at successively lower positions are transcribed later (Röttinger et al., 2012).

Our data enable us to add detail to the *Nematostella* gene regulatory network described by Röttinger et al. (2012). The widespread impacts on gene expression in *Nvbra*/Cas9 embryos argue that *Nvbra* has a central role in this GRN in early embryos. Disrupting expression of the transcription factor Nvbra has effects both within and outside its expression domain (Fig. 6). This suggests that *Nvbra* acts directly on genes within its expression domain, and triggers signaling events that affect gene expression in nearby cells.

Specification of endodermal cells in *Nematostella* is initially dependent on canonical Wnt signaling (Röttinger et al., 2012), specifically the nuclearization of β-catenin in cells of the animal pole at the 16-32 cell stages (Wikramanayake et al., 2003; Lee et al., 2007; Leclère et al., 2016). [Endodermal specification also relies on inputs from BMP (Wijesena et al., 2017) and FGF (Layden et al., 2016; A. R. Amiel, H. Johnston, T. Chock, P. Dahlin, M. Iglesias, M. J. Layden, E. Rottinger and M.Q.M., unpublished) pathways.] As *Nvbra* expression precedes that of Wnt genes (Röttinger et al., 2012), this suggests that *Nvbra* expression is an early response to nuclear β-catenin and that *Nvbra* plays a very early role in the GRN.

Röttinger et al. (2012) showed that inhibiting the Wnt pathway (by expressing *Nvdntcf*, a dominant-negative *Nvtcf*) in *Nematostella* strongly downregulates *Nvbra*. Because we see downregulation of *Wnt* gene expression in *Nvbra*/Cas9 embryos, this suggests that a *Nvbra*-*Wnt* feedback loop operates in early *Nematostella*; a similar feedback loop has been documented in zebrafish (Martin and Kimelman, 2008). Some genes show similar responses to both *Nvdntcf* and *Nvbra* gene excision (e.g. *Nvgsc*, *NvFGFa1*, *NvfoxA*, *Nvwnt2*, *Nvwnt3* and *Nvwnt4*), suggesting that some downstream effects of *Nvbra* are mediated though its activation of Wnt expression. In contrast, numerous genes show opposing responses to the two perturbations (e.g. *NvfoxQ1*, *Nvtolloid*, *Nvsprouty* and *NvotxA*), indicating that not all of the effects of *Nvbra* are mediated by Wnts. Together, these results argue that, while *Nvbra* appears to be necessary for expression of several Wnts, it does not act solely though the Wnt signaling pathway.

### Effects of *Nvbra* excision on patterning of the oral-aboral axis

*Nvbra*/Cas9 embryos fail to elongate after gastrulation and remain almost spherical, likely due to the absence of pharyngeal elongation. Some aspects of ectodermal cell differentiation appear to occur normally, including formation of an apical tuft and development of cnidocytes (Fig. 3). Expression of some region-specific markers along the oral-aboral axis, such as *NvFGFa1* and *Nvtbx4/*5, was altered (Fig. 5). Given that *Nvbra* excision dramatically affects expression of five distinct Wnt genes (Figs 4 and 5), the effects on the oral-aboral axis are likely to be mediated by disrupted Wnt signaling, as several lines of evidence indicate that Wnt proteins pattern the embryo along this axis at postgastrula stages (reviewed by Rentzsch and Technau, 2016).

Finally, *Nvbra* excision leads to radialized expression of *NvBMP2/4*, a gene that shows asymmetric expression during early gastrulation, and which is involved in determining the directive axis (Finnerty et al., 2004; Matus et al., 2006; Saina et al., 2009). Two modulators of BMP signaling (*Nvkielin*, *Nvtolloid*) are also upregulated, suggesting that *Nvbra* may be involved in specifying the directive axis as well as the oral-aboral axis.

### Evolutionary role of *brachyury*

The *brachyury* gene originated in the opisthokont ancestor (Sebé-Pedrós et al., 2013). Recent findings in the filasterean *Capsaspora* suggest that, among other roles, *brachyury* controls a number of genes involved in cell motility (Sebé-Pedrós et al., 2016). In metazoans, *brachyury* is expressed at sites of cell movements – the blastopore and, in many cases, the forming stomodeum/pharynx – in organisms including ctenophores, cnidarians, protostomes and deuterostomes (Satoh et al., 2012). Although *brachyury* is expressed around the blastopore, its precise role there is not clear, as animals lacking *brachyury* function can still undergo at least the initial stages of gastrulation and endoderm specification. These data suggest that an ancestral metazoan role of *brachyury* was not to specify mesoderm per se, but to delimit a boundary of differential cell behavior and motility between germ layers, although this role was clearly co-opted in some lineages (e.g. for notochord formation) later in evolution.

The results of Yasuoka et al. (2016) support this view; they showed that loss of *brachyury* in *Acropora* results in the absence of the pharynx, and concluded that the gene has an evolutionarily conserved role in morphogenesis and cell motility. They speculate that chordate mesoderm may have an ectodermal origin. An alternative view, that mesoderm likely originated from endoderm, is based on two observations, although each has caveats. First, a transient bipotential endomesodermal region is specified in many organisms. Nuclear β-catenin is thought to represent an ancient mechanism for such specification; the endomesodermal region is subsequently segregated into endodermal and mesodermal precursors (Kimelman and Griffin, 2000; Rodaway and Patient, 2001; Schneider and Bowerman, 2013; Darras et al., 2011; Hudson et al., 2013, 2016; Logan et al., 1999; McCauley et al., 2015; Sethi et al., 2012). Similarly, definitive muscle cells arise from endomesodermal precursors in both ctenophores (Martindale and Henry, 1999) and acoel flatworms (Henry et al., 2000), both of which branch before the protostome-deuterostome divergence, suggesting that mesoderm evolved from endoderm. However, muscle may not be a definitive indicator of the origin of mesoderm, as striated muscle may have evolved independently in cnidarians and bilaterians (Steinmetz et al., 2012). Second, several genes associated with mesoderm are expressed in cnidarian endoderm (Martindale et al., 2004), suggesting that mesoderm segregated from endodermal, not ectodermal, precursors during evolution. On the other hand, two bilaterian mesoderm genes, *brachyury* and *mef2*, are expressed in cnidarian ectoderm (Martindale et al., 2004). Although the evolutionary origin of mesoderm remains controversial, we favor the idea that mesoderm arose from endoderm, and that *brachyury* marks the limit of the endoderm in cnidarians and other systems, although it plays crucial roles in the normal development of both its own expression domain and of surrounding tissues.

### Conclusions

Recently, Ikmi et al. (2014) and Kraus et al. (2016) showed that Cas9 can mediate gene editing in *Nematostella*. We have used Cas9 to efficiently excise genes from early embryos, establishing this as a valuable tool for exploring gene function during early *Nematostella* development. Our data further show that *Nvbra* is necessary for development of the pharynx, and affects endodermal patterning and allocation of cells along the oral-aboral axis; we have also been able to establish connections among genes in the endomesodermal gene regulatory network (Röttinger et al., 2012).

## MATERIALS AND METHODS

### Embryos

*Nematostella vectensis* adults and embryos were cultured at 16°C in the dark in 1/3× filtered sea water (FSW). Animals were fed freshly hatched brine shrimp once or twice per week. Two to 5 days prior to spawning, animals were fed minced oyster. Spawning was induced by placing the animals at 25°C and exposing them to bright light for 8-9 h; they were then placed at room temperature in ambient light, where they spawned within 2-3 h. Eggs and sperm were mixed for 10-20 min, fertilized eggs were dejellied in 4% L-cysteine in 1/3× FSW, then washed three times in 1/3× FSW. Embryos were transferred to plastic petri dishes in 1/3× FSW for injection.

### Guide RNAs (gRNAs)

Target sites were identified using the ZiFit Targeter (http://zifit.partners.org/ZiFiT/ChoiceMenu.aspx). We designed oligonucleotides according to Varshney et al. (2015) and Gagnon et al. (2014). Briefly, these consist of a T7 promoter, followed by the 20-base target sequence [targets were chosen to start with GG, to maximize transcription by T7 polymerase (Gagnon et al., 2014)] and a 20-base sequence complementary to a second oligo (Table S1); the second oligo is the same for all reactions and contains the tracrRNA sequence. The two oligos were mixed, PCR-amplified, purified with a PCR purification kit (Qiagen) and transcribed *in vitro* (NEB HiScribe T7 high-yield RNA synthesis kit). RNA was purified with a spin column (Zymo), quantified (Qubit), concentrated (Speed-Vac) and frozen at −80°C.

### Analysis of possible off-target sites

We analyzed gRNA target sequences to identify possible off-target sites (OTSs) in the *Nematostella vectensis* genome using CCTop (Stemmer et al., 2015). A total of 53 OTSs containing four or fewer mismatches were identified for the five gRNAs. Of these, 51 contained four mismatches. Both OTSs containing three mismatches had two mismatches within the PAM-proximal 12 bases.

### Microinjections

Lyophilized Cas9 (PNA Bio) was reconstituted in 50% glycerol and 0.1 mM DTT. Embryos were injected as described previously (Layden et al., 2013) with a mixture containing gRNAs (80 ng/μl of each gRNA), Cas9 (1 μg/μl), and Alexa Fluor 488-dextran (0.2 μg/μl, Molecular Probes).

### Analysis of genomic DNA

Genomic DNA was extracted as described previously (Ikmi et al., 2014), except that we used 0.5 μg/μl proteinase K. Single embryos were transferred to 200 μl PCR tubes, and as much FSW was removed as possible. DNA extraction buffer with proteinase K was added, tubes were vortexed briefly, and samples were incubated for 2-3 h at 55°C with occasional vortexing. Proteinase K was inactivated for 5 min at 98°C. Each 25 μl PCR reaction contained 4 μl of extract. Genomic DNA was amplified with PCR primers flanking the targeted region (Fig. S1).

### *In situ* hybridization

Embryos were fixed and processed for *in situ* hybridization as described by Wolenski et al. (2013), except that embryos were fixed in *in situ* hybridization fixative 1 for only 90 s, and, after PTw washes, embryos were washed once in 100% methanol, then stored in 100% methanol.

### Immunohistochemistry

Immunohistochemistry was performed as described by Salinas-Saavedra et al. (2015) and Babonis et al. (2016). In brief, embryos were relaxed in MgCl_2_, fixed for 1 min at 25°C in 4% paraformaldehyde and 2.5% glutaraldehyde in PTw (PBS with 1% Tween), and for 1 h at 4°C in 4% paraformaldehyde. Fixative was removed and embryos were washed three times (5-15 min each) in PTw and stored in PTw at 4°C before processing (up to 1 month). Tissues were rinsed in three washes (15-30 min each) of PBT (PBS, 1% bovine serum albumin, 1% Triton X-100). Non-specific protein interactions were blocked for 1 h at 25°C in 5% normal goat serum (NGS) in PBT. NGS/PBT was removed and replaced with a primary antibody in NGS/PBT: either anti-minicollagen 4 diluted 1:1000 (Babonis et al., 2016), anti-aPKC (1:100) or anti-Lgl (1:100) (Salinas-Saavedra et al., 2015). Tissues were incubated overnight (∼12-18 h) at 4°C. Tissues were washed three times (15 min each) in PBT, then incubated for 1-2 h at 25°C in secondary antibody (goat anti-rabbit-647; Invitrogen A21245) (1:500 in PBT). Embryos were washed at least three times in PTw (15 min each) at 25°C. Cell membranes (f-actin) and nuclei were simultaneously labeled by reconstituting fluorescent phalloidin (Invitrogen A12379) at 1:200 in a solution of 2 ng/ml propidium iodide (Sigma 81845) and 2 ng/ml RNase A (Sigma R5000) in PTw and incubating overnight at 4°C. Embryos were rinsed with at least three washes (15 min each) of PBS, dehydrated through a graded isopropanol series (70-100%), then cleared/mounted in 2:1 benzoic acid: benzoyl benzoate. Confocal microscopy was performed using a Zeiss 710 LSM at the Whitney Marine Lab (University of Florida, St Augustine, FL, USA). Images were artificially brightened using Adobe Photoshop and are presented as single optical sections from confocal *z*-stacks unless otherwise noted. 3D reconstructions of embryos were rendered from *z*-stacks using Imaris (Bitplane).

### Quantitative PCR (qPCR)

Three (24 h) or 4 (48 h) samples from separate rounds of injection were analyzed separately. qPCR was carried out using a LightCycler 480 (Roche) with SYBR Green Master Mix, as described by Layden et al. (2012). Reactions for each gene, at each time point, were carried out in triplicate. Ribosomal protein P0 was used as a control to normalize RNA levels (Peres et al., 2014). Reductions in expression are shown as the negative reciprocal of the expression level, to facilitate visualization.
